# PAIRS: Prediction of Activation/Inhibition Regulation Signaling Pathway

**DOI:** 10.1155/2017/7024516

**Published:** 2017-04-02

**Authors:** Tengjiao Wang, Yanghe Feng, Qi Wang

**Affiliations:** ^1^Second Military Medical University, Shanghai, China; ^2^Science and Technology on Information Systems Engineering Laboratory, National University of Defense Technology, Changsha, Hunan, China

## Abstract

Uncovering the signaling architecture in protein-protein interaction (PPI) can certainly benefit the understanding of disease mechanisms and promise to facilitate the therapeutic interventions. Therefore, it is important to reveal the signaling relationship from one protein to another in terms of activation and inhibition. In this study, we propose a new measurement to characterize the regulation relationship of a PPI pair. By utilizing both Gene Ontology (GO) functional annotation and protein domain information, we developed a tool called Prediction of Activation/Inhibition Regulation Signaling Pathway (PAIRS) that takes protein interaction pairs as input and gives both known and predicted result of the human protein regulation relationship in terms of activation and inhibition. It helps to give prognostic regulation information for further signaling pathway reconstruction.

## 1. Introduction

The rapid increase in genomic information requires new techniques to infer protein function and predict protein-protein interactions. To properly understand normal cellular responses and their potential dysregulation in disease, a global multivariate approach is required [1]. Many studies, using machine learning methods, have been carried out to investigate the regulation signaling pathway. Bayesian network method was used in [2] to predict novel pathway network causalities. Yaffe et al. proposed a peptide library-based searching algorithm and improved the searches for proteins containing motifs matching two different domains in a common signaling pathway [3]. Hill et al. [4] incorporated existing biology using informative network priors, weighted objectively by an empirical Bayes approach, and exploit a connection between variable selection and network inference to enable exact calculation of posterior probabilities of interest. With the help of the computational methods these studies elucidated most of the traditionally reported signaling relationships. Bioinformatics tools are treated as the right arm of the signaling pathway study since they promise a quick interpretation of OMICS data. Software was designed based on different purpose. A universal sequence relation drawing program was developed for accelerating translational bioinformatics research in [5]. Karp et al. [6] provide an overview of the four main components of the pathway tools. PathoLogic was developed to create a new pathway genome database containing the predicted metabolic pathways of an organism when given a GenBank entry as input; Pathway/Genome Navigator was developed to support query, visualization, and analysis of pathways. The Navigator powers the BioCyc web site; MetaFlux was developed to support the development of metabolic flux models. Some of the methods and software focus on a particular biological functional pathway or an integrated database. Some studies were dedicated to developing a more efficient statistical inference method. With the boom in machine learning methods and high credibility biological database [7], new methods are in great need to help identify novel pathway regulation relationships of protein interactions.

**Figure 1:**
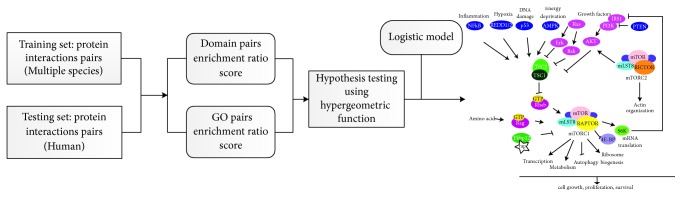
The workflow of PAIRS.

In this paper, we first integrated GO and protein domain information. Then we proposed the enrichment ratio (ER) score for each GO term or domain term interaction pair; for one activation/inhibition relationship of a protein interaction pair, the ER score was treated as the feature to distinguish the regulation relationship in terms of activation/inhibition. By using the ER score as new feature, we developed a web tool, PAIRS, to give extensive prediction of protein pathway regulation relationship. The workflow of PAIRS is shown in Figure 1.

## 2. Materials and Methods

### 2.1. Extraction of GO and Domain Information

As a standard terminology in genome research, Gene Ontology (GO) [8] covers three domains: cellular component, molecular function, and biological process. In a pathway, one protein regulation usually related to several GO terms. Therefore, the GO annotation is an important data source to infer new interactions and regulation relationships in a signaling pathway. The GO data was downloaded from http://geneontology.org/page/downloads.

Considered as the conserved part of a protein, domains are independently stable. One domain may appear in a variety of different proteins. Different domain combinations give rise to the diverse range of proteins found in nature. Therefore, protein domain information is also an important data source to infer new regulation relationships in a signaling pathway. Here, the PFAM database [9] was used to extract the protein domain information.

### 2.2. Computing the Enrichment Ratio Score as New Feature

The enrichment ratio was first posed by Liu and Xie [10]. It was used to investigate the enrichment extent of a domain pair appearing in the activation dataset or inhibition dataset. The enrichment ratio (ER) is defined as

(1) $$\begin{array}{c} ER=\frac{m/M}{n/N}, \end{array}$$

where *N* is the protein interactions number in the whole standard dataset and *M* is the protein interactions number in the activation/inhibition dataset. For a specific pair of domains, *n* is defined as the number of protein interactions containing this pair in the whole standard dataset and *m* is the number of protein interactions containing this pair in the activation/inhibition dataset.

The hypergeometric test was adopted to measure the statistical significance of ER. The hypothesis test was designed to investigate the overrepresentation of GO or domain pairs appearing in the activation/inhibition dataset. The hypergeometric *P* value is calculated as the probability of randomly drawing *m*′ or more successes from the population in *n* total draws. For ER ≥ 1 the *p* value is defined as

(2) ∑m′=mnPX=m′∑m′=mnfm′;N,M,n=∑m′=mnMm′N−Mn−m′Nn

and for ER < 1 the *p* value is defined as

(3) ∑m′=0mPX=m′∑m′=0mfm′;N,M,n=∑m′=0mMm′N−Mn−m′Nn.

### 2.3. Preparing Training and Testing Dataset

All signaling networks were extracted from KEGG (Kyoto Encyclopedia of Genes and Genomes) [11]. The regulation relationship in terms of activation and inhibition usually stays the same in human species as well as in other species. Therefore, the regulation relationships from multiple species tend to be more credible. Here, 1893 protein interactions shared in multiple species (human, rat, mouse, fly, and yeast) were used as training set. Among them, 1554 protein interactions' regulation relationships are activation; 339 interactions' regulation relationships are inhibition.

Other protein interactions with activation/inhibition regulation information were obtained from Liu et al. [12]. There are 6,791 protein regulation pairs with 5,261 activation interactions and 1530 inhibitions. Excluding those interactions in the training set, the rest was used as the independent test to identify novel regulation relationships. The human protein interactions were extracted from HPRD, DIP, MINT, and BIND database and previous resources [13, 14].

### 2.4. Predicting of Regulation Relationship of Signaling Pathway with Logistic Regression

Several machine learning techniques have been adopted for prediction in the domain of bioinformatics [4, 15–18]. As suggested in study [10], after the training and testing dataset were prepared, we adopted the logistic regression method to conduct the training and predicting step. The logistic regression is a type of probabilistic statistical classification model, which is always used for predicting the outcome of a categorical dependent variable (i.e., a binary class label) based on one or more predictor variables (features). The logistic regression function can be written as

(4) $$\begin{array}{c} F\left(\;x\;\right)=\frac{1}{1+e^{-\left(\beta_{0}+\beta_{1}x\right)}}, \end{array}$$

where *x* is explanatory variable as the vectors of significant ER in GO or domain pairs. *F*(*x*) is confined to values between 0 and 1 and hence is interpretable as a probability of regulation relationship being activation (or inhibition). Then the other regulation relationship inhibition (or activation) is 1 − *F*(*x*). Here, *β*_0_ is the intercept from the linear regression equation and *β*_1_ is the regression coefficient multiplied by some value of the predictor. *β*_0_ and *β*_1_ are the regression coefficients. In the binomial logistic regression model with L1 regulation, the beta 1 is the sparse vector with more than 73689 dimensions. The WEKA package [19] was used to perform the binomial logistic regression on the extracted training set of the regulation relationships to build the classifier. fivefold cross-validation was used for evaluation. We also test the L2 regulation with the same training data. The 5-fold cross-validation shows the lower precision and recall ratio than L1 regulation. The trained classifier was performed on the testing dataset. Some regulation relationships in known regulation signaling pathway were checked to inspect the performance of our method. Based this model, we provide a server/client tools which work on-line or off-line modes (the client can be downloaded at https://fengyanghe.github.io/). It can be used to identify the regulation relationships by estimating the ER score of GO or domain pairs.

Before PAIRS predicts the regulation relationship of the input protein-protein interaction pair, it will check if regulation relationship of the input pair is already known. If it is known, then PAIRS will output the known regulation directly.

## 3. Results and Conclusions

**Figure 2:**
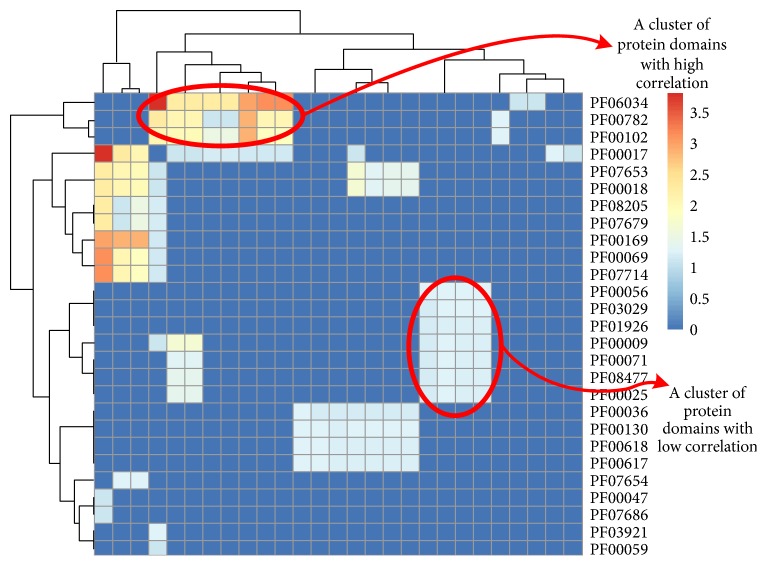
The heat map of ER from apoptosis domain interaction.

After the ER is computed, in a certain pathway some of the GO or domain interaction pairs may be correlated. For example, in the known apoptosis pathway as shown in Figure 2, some domains have high ER (red colour) while most remain low (blue colour) and domains can be hierarchically clustered by their ER. It reveals that, in a pathway, the protein regulation is always involved with correlated domain pairs. The same results also apply to GO terms.

Only the nonredundant interactions with corresponding Entrez Gene ID and not reported in protein complex were extracted from the human proteome-wide interaction dataset, which is mentioned before. The total number of protein interactions is 45,238. As shown in Table 1, some known human signaling pathways were used to test the performance of the trained classifier, after the logistic regression model was fitted by the training dataset. The accuracy of the classifier which used GO terms ER as features is slightly lower than the accuracy of the classifier which used domain terms ER as features. This is because the domains are the units of protein structure and evolutionary modules; it directly reflects on the combining feature of interaction itself. GO is the functional interpretation of a protein. When used as interaction properties to infer the regulation relationship in a pathway, domain pairs performed better than GO pairs. However, the GO term pairs usually exist in more protein-protein interactions than the domain pairs. Therefore, the coverage of prediction results is larger when the ER score was computed by GO pairs than by domain pairs.

**Figure 3:**
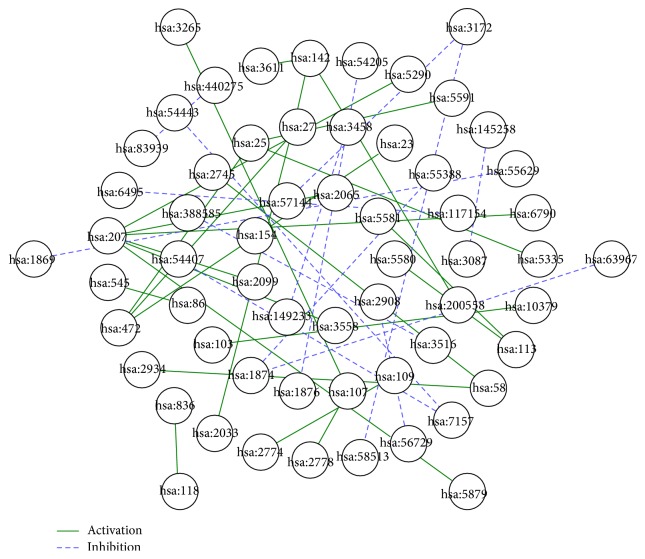
An example of the output result by PAIRS.

We developed a web tool, called PAIRS, to identify the regulation relationships. The input of PAIRS is protein interaction pair. After GO or PFAM was chosen, the ER score of GO or domain pairs are computed by PAIRS to construct the feature vector for each protein-protein interaction pair. Then, the trained classifier was used to predict the regulation relationship of the protein interaction pairs. As shown in Figure 3, the solid line denotes the regulation relationship as activation, the dash line denotes the regulation relationship as inhibition. The size of protein node is proportional to their degree.

For a certain protein-protein interaction, PAIRS outputs the interaction type and it also gives the significant ER scores of GO or domain pairs that was used.

## 4. Discussions

The challenge of systematic approach requires the protein networks involving all protein-protein interactions and the metabolic networks involving all enzymes and pathways. Bioinformatics methods can be used to accelerate the discovery of regulation relationship between protein interactions and distinguish the activation relations from inhibition relations. Reconstruction of signaling networks from protein interactions might be applied to understanding signaling transduction process, complex drug actions, and dysfunctional signaling in diseased cells [20]. In this study, we developed PAIRS to infer the regulation relations of protein interactions. Using GO terms and domain interaction dataset, PAIRS computes the novel indicator (ER) to construct the feature vector and utilizes the logistic regression to predict regulation relationships in human pathway. Then we evaluated the performance of PAIRS on protein interactions in known signaling pathway. The prediction results together with the corresponding GO or domain pairs which are used in the prediction are provided by PAIRS.

The limit of PAIRS lies in that if the interacting proteins do not contain either domain or GO interaction pairs, PAIRS cannot give any results. And if the inputs contain a large amount of protein interactions, the prediction computational time by the classifier will increase. With the development of efficient machine learning method and comprehensive biological data sources, PAIRS can be improved.

## Figures and Tables

**Table 1:** The prediction results of the method in known human signalling pathways.

Signaling pathways	Accuracy of GO (%)	Accuracy of domain (%)
MAPK signalling pathway	100	100
T cell receptor signalling pathway	100	100
VEGF signalling pathway	100	100
Wnt signalling pathway	93.32	96.97
TGF-beta signalling pathway	81.53	87
mTOR signalling pathway	70.44	75
